# Digestions

**Published:** 1842-06-18

**Authors:** 


*Digestion.*—Some kinds of animal food are digested with much greater rapidity than vegetable food. Bread and coffee require above 4¼ hours; fresh beef, from 3 to 3½ hours; salt beef, from 3½ to 5½; salt pork, from 4½ to 6; mutton, 3¼ to 4½; fowl, 4; veal, from 4 to 5½; tripe, 1; and pig's feet, 1.—*Brit. and For. Med. Rev.*

